# Solar Drying for Domestic and Industrial Applications: A Comprehensive Review of Innovations and Efficiency Enhancements

**DOI:** 10.1002/gch2.202400301

**Published:** 2025-01-12

**Authors:** Md Atiqur Rahman, S. M. Mozammil Hasnain, Prabhu Paramasivam, Rustem Zairov, Abinet Gosaye Ayanie

**Affiliations:** ^1^ Department of Mechanical Engineering Vignan's Foundation for Science Technology & Research Vadlamudi Guntur Andhra Pradesh 522213 India; ^2^ Marwadi University Research Center Department of Mechanical Engineering Faculty of Engineering & Technology Marwadi University Rajkot Gujarat 360003 India; ^3^ Kazan Federal University 1/29 Lobachevskogo Str. Kazan 420008 the Russian Federation; ^4^ A.E. Arbuzov Institute of Organic and Physical Chemistry Kazan Scientific Center, Russian Academy of Sciences 8 Arbuzov str., Kazan, Russian Federation R 420088 Russia; ^5^ Department of Mechanical Engineering Adama Science and Technology University Adama 2552 Ethiopia

**Keywords:** direct solar dryer, hybrid solar dryer, indirect solar dryer, photovoltaic, solar absorber plate

## Abstract

Global challenges such as energy scarcity and food security are intensified by a growing population and substantial post‐harvest food losses, contributing to alarming hunger levels. Solar drying is recognized as an effective, high‐quality, and sustainable method for food preservation, significantly aiding global food security. Dryers are essential in agriculture and the food industry for extending crop shelf life by removing moisture through thermal energy, with solar thermal energy being particularly suitable due to its environmental benefits and availability. This article reviews the classification of solar dryers, including direct (DSD), indirect (ISD), and hybrid (HSD) systems, examining key components like solar collectors, drying chambers, and auxiliary systems and the factors affecting their performance. The review highlights that the efficiency of solar dryers depends on dryer type, solar irradiation, drying duration, and operational conditions. Recent advancements to enhance solar dryers' energy efficiency include hybrid systems incorporating auxiliary heating sources (electric or biomass), solar‐assisted heat pump dryers, surface modification techniques, and heat storage systems utilizing sensible and latent heat storage. Findings suggest that HSD with auxiliary units can achieve up to 54% efficiencies, while solar collectors can reach up to 81%, yielding better product quality than traditional ones.

## Graphical abstract

Innovations advancing solar drying for sustainable food preservation and industrial applications.

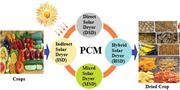

## 1 Introduction

In the present circumstances, with the global population surpassing the threshold of 8 billion individuals,^[^ ^1^ ^]^ the increased demands for energy and food have emerged as significant concerns.

In 2021, the percentage of food lost globally after harvest on farm, transport, storage, wholesale, and processing levels was estimated at 13.2 %.^[^ ^2^ ^]^ In 2022, food waste occurring at retail, food service, and household levels was estimated to be 19 % of all food available to consumers.^[^ ^3^ ^]^

**Figure 1:**
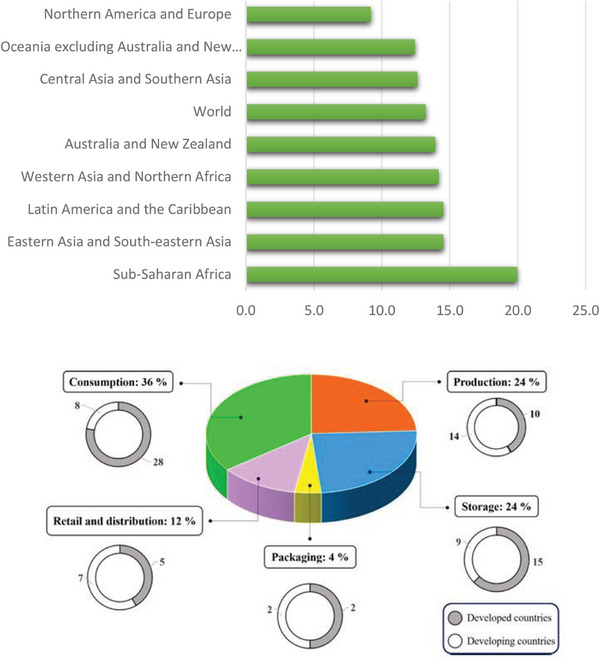
(a) Food loss percentage by region in 2021 Reproduced with permission.^[^ ^2^ ^]^ (b). Global food waste distribution from production to consumption. The donut charts demonstrate the food waste in developing and developed countries (%) across the value chain. Reproduced with permission^[^ ^5^ ^]^ Copyright 2021 Elsevier Ltd.

Food loss and waste undermine the sustainability of our food systems. When food is lost or wasted, all the resources that were used to produce this food – including water, land, energy, labor, and capital – go to waste. In addition, the disposal of food loss and waste in landfills leads to greenhouse gas emissions, contributing to climate change. Food loss and waste can also negatively impact food security and availability and increase food costs. Minimizing food losses and waste is crucial as global hunger has steadily increased since 2014, while significant quantities of edible food are lost or wasted daily; additionally, substantial losses of food post‐harvest present auxiliary challenges for society. The concept of “post‐harvest food losses” pertains to the reduction in quantity and quality of various food items from the point of harvest through to final consumption. When considering losses at retail and consumer levels, this percentage increases to ≈32.2%. **Figure** **1**a indicates that the global food loss rate remained steady, from 13 % in 2016 to 13.2 % in 2021. Regions experiencing an increase in food loss rates include Latin America and the Caribbean, with the most significant rise from 12.2 % in 2016 to 14.5% in 2021, as well as in Western Asia, Northern Africa, Australia, and New Zealand.

This data concerns a world where ≈735 million people are undernourished and struggling to obtain sufficient food.^[^ ^4^ ^]^ Indeed, the amount of food lost or wasted annually could feed ≈1.26 billion hungry people.^[^ ^2^ ^]^ Further, Figure 1b provides a detailed breakdown of global food waste across developing and developed nations, encompassing percentages allocated to consumption, production, storage, packaging, retail, and distribution stages. Inefficient stock planning contributes significantly to market overstocking, driven by consumer preferences for less popular items and more significant food portions, leading to substantial leftovers. Consumer behavior, such as reluctance to take leftovers or discarded food, further exacerbates this issue. The distribution stage involves distribution centers, wholesalers, retailers, and associated transport networks. Perishable goods are particularly prone to wastage during storage and distribution processes.

An essential cause of fruit & vegetable spoilage is microorganisms. The sources of microbes include faecal matter, transport rot, Earwina, Pseudomonas, water and soil pathogens, yeast, and molds on the vegetable surface. Reports state that physical contamination, insect, and dust processing conditions, where in storage temperature, act like a booster. Gram‐negative bacterial flora causes major spoilage of vegetables. These spoilage organisms thrive and multiply faster at ambient temperatures and high humidity.^[^ ^6^ ^]^ These observations underscore the necessity for implementing efficient food drying techniques, which are crucial for promoting sustainable agriculture. Such strategies are essential to achieving the United Nations Sustainable Development Goals (SDGs), specifically SDG 2 of zero hunger, by ensuring enhanced food security through more effective means.^[^ ^7^ ^]^

Drying or “dehydrating” food is a method of food preservation that removes enough moisture from the food so bacteria, yeast, and molds cannot grow. In recent years, solar drying systems have emerged as one of the foremost effective and high‐quality strategies for food processing.^[^ ^8^, ^9^, ^10^ ^]^

Solar dryers use solar energy to dehydrate substances, mainly food. In contrast to traditional sun drying methods, where food items are exposed directly to sunlight in an open environment, solar drying employs indirect solar radiation. The fundamental principle of solar drying involves using solar collectors to capture and heat air, which is then circulated into an enclosed drying chamber. Within this chamber, the food products intended for drying are placed systematically. This method offers advantages over sun drying, such as improved control over environmental variables and reduced risk of contamination, making it suitable for larger‐scale food processing operations.^[^ ^11^, ^12^, ^13^ ^]^

The initial prototype of a solar food dryer was conceived and constructed by Stanley and Colo in 1976.^[^ ^14^ ^]^ After its inception, continuous technical enhancements have been made to enhance its efficiency, facilitating its adoption across various domestic and industrial applications.^[^ ^15^, ^16^, ^17^, ^18^, ^19^ ^]^ **Table** **1** compiles recent studies that explore the qualitative aspects of solar‐dried products, focusing mainly on the retention of nutritional values.

**Table 1:** Recent studies focusing on the quality achieved through solar drying applications.

Author	Solar dryer Type	Sample [Fruit/crop] used	Qualitative conclusions
Kumar et al.^[^ ^20^ ^]^	Evacuated tube solar dryer	okra pods	The maximum retention of chlorophyll content (76.70 to 93.20%), vitamin C (73.84 to 93.73%), and minimum color changes (7.70 to 13.48) as compared to OSD
Dutta et al.^[^ ^21^ ^]^	Evacuated tube solar dryer with PCM	Turmeric	retention of curcumin contents (7.49%), antioxidants (65.92 %), and TPC (22.38 mg GAE g^−1^), respectively, compared to OSD
Tchicaya et al.^[^ ^22^ ^]^	solar‐assisted heat pump dryer with a TES system	Cavendish banana	Nutrient proportion retention of 80.1% as compared to fresh Cavendish banana 21.6%
Afzal et al.^[^ ^23^ ^]^	hybrid mix‐mode solar dryer	Freestone peach, Golden apple, Anaheim chillies	The result indicates a low percentage of ash, crude protein, fat, and fiber and a high percentage of carbohydrates.
Roratto et al. ^[^ ^24^ ^]^	Hybrid solar vacuum dryer	Banana, Persimmon, Carrot	The dried samples were uniform in texture, crisp, and showed higher nutritional values.
Lehmad et al.^[^ ^25^ ^]^	Hybrid Solar‐Electrical Drying	Larvae of black soldier fly.	Retain vales in protein (g/100 g) of 38.14, Lipid (g/100 g) of 21.24, ash (g/100 g) of 7.35, pH of 9.25.
Catorze et al. ^[^ ^26^ ^]^	A hybrid solar dryer with a fan running in PV support is needed.	Blueberries, Raspberries, Spinach	Considerable nutritional retentions were seen in the case of dried samples, including antioxidants, proteins, TPC, and sugar.

Solar dryers offer several key advantages over OSD, primarily focusing on reduced drying times, cost‐effectiveness, increased efficiency, enhanced hygiene, and healthier final foodstuffs. These systems harness solar energy, which is used for drying in a controlled environment, resulting in improved product quality with decreased losses(post‐harvest).

## 2 Types of Solar Dryers

**Figure 2:**
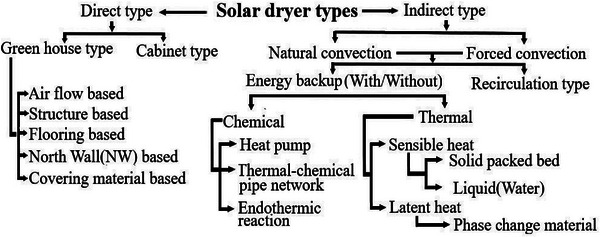
Classification of solar dryers.

Researchers worldwide have developed diverse types of solar dryers, which can be categorized based on design, construction materials, auxiliary heat sources, dried substances, and TES (Thermal energy storage) material. **Figure** **2** illustrates the general classification of solar dryers.^[^ ^27^ ^]^

Solar drying involves two distinct stages: initially, solar radiation facilitates the transfer of thermal energy from the sun to the drying material(Heat transfer), and subsequently, moisture from the drying material is transferred into the surrounding environment as vapor (mass transfer). Based on the method of heat transfer, solar dryers can be categorized as direct, indirect, or mixed‐mode types.^[^ ^28^ ^]^ Furthermore, based on how moisture is transferred, solar dryers can be categorized as either natural or forced convection systems.^[^ ^29^ ^]^ A vital aspect of this process is the drying air, which is hot and low in moisture content. Additionally, the drying rate increases with higher temperatures and air velocities.

**Figure 3:**
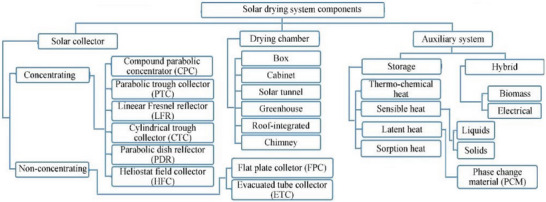
Components of solar dryer.

Drying, the oldest food preservation method has historically utilized solar energy. Solar drying can occur outdoors, known as Open Sun Drying (OSD), where food is spread on a clean surface under the sun or in specially designed solar dryers. OSD, however, has limitations such as susceptibility to spoilage from rain, moisture carried by wind, and contamination from dust, leading to risks of fungal, bacterial, and insect damage.^[^ ^30^, ^31^ ^]^ In contrast, solar dryers offer several advantages over OSD. First, they are more efficient, requiring less time and space for drying. Second, they protect the product from rain, insects, animals, and dust, which may contain contaminants. Thirdly, faster drying reduces the risk of mold growth. Fourthly, higher drying temperatures facilitate more thorough drying, extending storage times significantly, provided humidification is prevented during storage.^[^ ^32^ ^]^ Numerous scholars, researchers, and reviewers have also classified solar dryers based on the working component in **Figure** **3**.

### 2.1 Basic Types of Solar Dryer

#### 2.1.1 Direct‐Type Solar Dryers

The direct solar dryer (DSD) is characterized by a straightforward, cost‐effective design that protects from dust, rain, dirt, and dew. In DSD, solar radiation directly dries the crops inside the drying chamber, typically enclosed with transparent materials such as glass or polyethene sheets. These materials absorb, transmit, and reflect different portions of the incoming radiation. The solar insolation that passes through the covering material becomes trapped inside the chamber, raising the internal temperature of the dryer. As the chamber temperature increases, moisture from the crop surface evaporates. However, DSD has drawbacks, including potential issues such as overheating, compromised product quality, and limited drying capacity.

**Figure 4:**
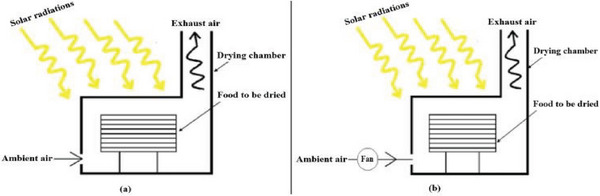
Types of Direct type solar dryers (a) natural convection. (b) Forced convection. Reproduced with permission^[^ ^34^ ^]^ Copyright 2024 Springer Nature.

These may be of natural convection type^[^ ^33^ ^]^ or forced convection (**Figure** **4**).^[^ ^34^ ^]^ A few of the natural convection‐direct solar dryers (NDSD) are discussed below, which mainly started with the work of Fournier and Guinebault,^[^ ^35^ ^]^ who tested shell‐type solar dryer to dry mango slices under natural convection and determined that the thickness of the fruit slice is a critical factor affecting drying time.

**Figure 5:**
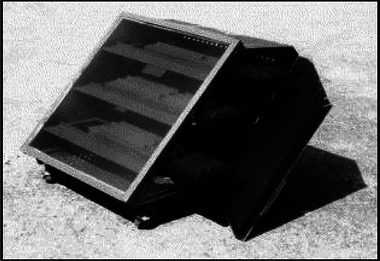
Conventional natural convection solar dryer Open door Solar dryer. Reproduced with permission Copyright 2005 Elsevier Ltd.^[^ ^37^ ^]^

**Figure 6:**
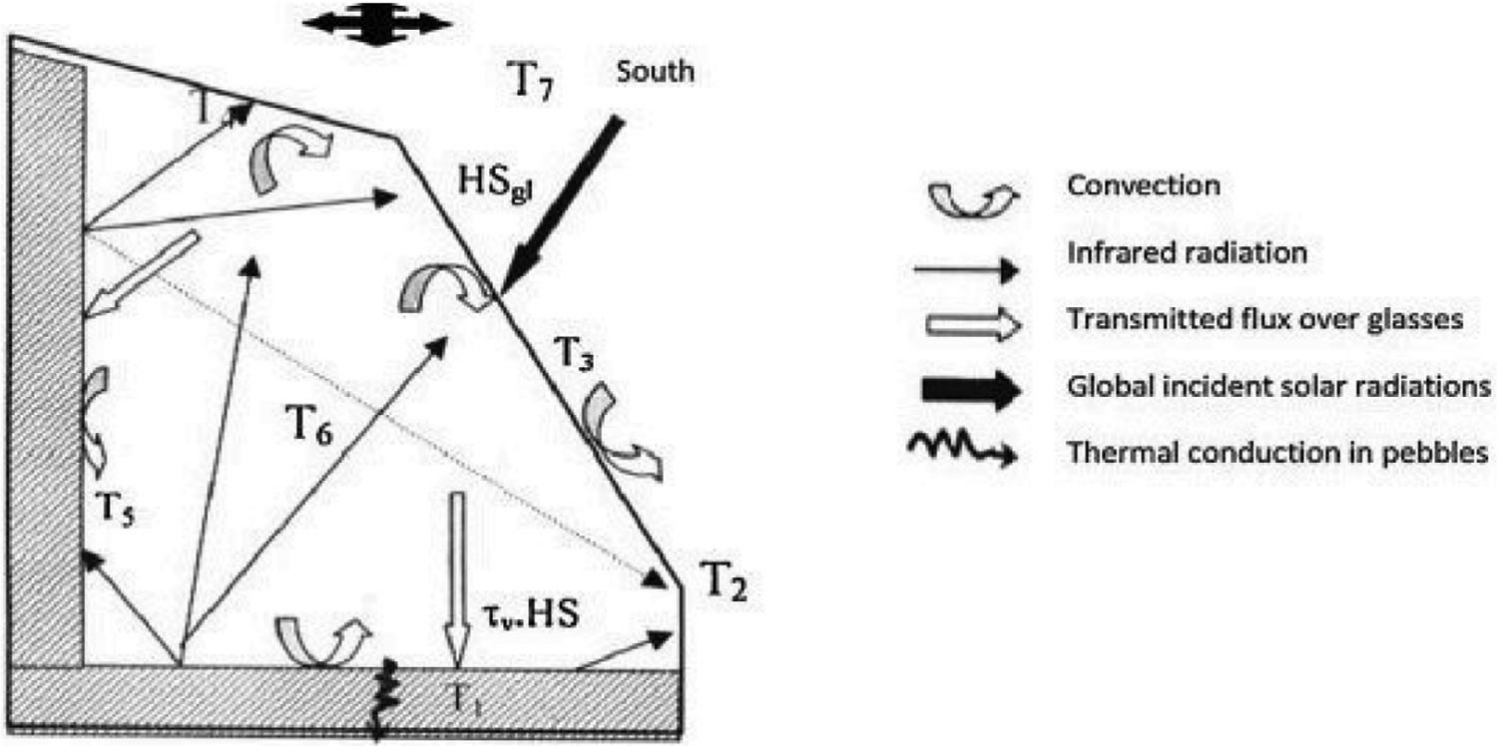
NDSD with pebbles.^[^ ^40^ ^]^

In NDSD mode, airflow within the crop is driven by buoyancy forces and augmented by wind pressure. Warmed air ascends naturally via the thermosyphon effect and is expelled through the greenhouse roof or designated ventilation points. These may be various types:

**Cabinet type**: Wakjira et al.^[^ ^36^ ^]^ designed and evaluated a closed‐system solar drying apparatus to determine the ideal banana slice thickness. The drying process spanned 4 days to achieve a moisture content deemed safe for slices measuring 3–4 mm, ensuring optimal quality. Singh et al.^[^ ^37^ ^]^ and Lawand.^[^ ^38^ ^]^ (**Figure** **5**) designed an NDSD comprising three distinct drying chambers, each featuring varied ventilation setups: thin tube chimney, attic, and simple configurations. Their experimentation involved drying apples, bananas, and guava. Among these, the simple‐type drying chamber demonstrated superior efficiency, achieving a maximum moisture removal rate of 58.9%, compared to 44.5% for the chimney type and 33.3% for the attic‐type ventilated drying chamber. Sharma et al.^[^ ^39^ ^]^ engineered and evaluated a cabinet‐style NDSD for drying peas, grapes, and potatoes. During testing, the maximum temperature of the trays reached ≈80 °C, which was significantly elevated, surpassing the ambient temperature by ≈50 °C. Mokretar^[^ ^40^ ^]^ introduced a new type of NDSD, as depicted in **Figure** **6**, with a base that included a layer of black‐painted pebbles utilized as a heat storage medium during adverse drying conditions. However, specific dimensions of the dryer were not provided. The solar dryer underwent testing in Alger from 7 July to 20 September 2010. Atmospheric conditions were stable, with air velocities ranging from 1.2 to 2 m s^−1^. The average temperature and radiation flux were measured inside the drying chamber at 46 °C and 653 W m^−^^2^, respectively. Various herbs and fruits, including mint, vervain, laurel, grape, prune, banana, fig, date, and pepper, were subjected to drying, with results that were compared against OSD methods. According to the authors, the drying process was significantly accelerated compared to OSD, achieving speeds ≈2 to 5 times faster. The cabinet dryer exhibited several drawbacks:

1. Limited material loading capacity, rendering it unsuitable for commercial use.
2. Lengthy drying times were required.
3. Reduction in glass cover transmissivity due to moisture evaporation and condensation.
4. Potential crop overheating from direct sunlight exposure, potentially degrading product quality.
5. Low efficiency is attributed to solar energy used for airflow induction, and the product acts as an absorber.

**Figure 7:**
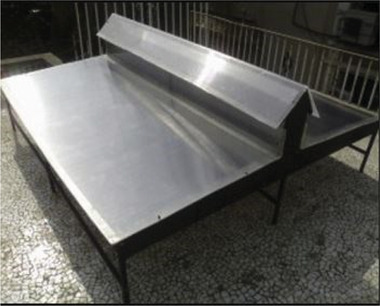
Chimney type NDSD solar conduction dryer. Reproduced with permission Copyright 2015 Elsevier B.V.^[^ ^41^ ^]^

**Chimney type**: Borah et al.^[^ ^41^ ^]^ conducted experiments on both whole and sliced turmeric samples to analyze drying kinetics, utilizing an NDSD depicted in **Figure** **7**. Bolaji^[^ ^42^ ^]^ devised an indirect NDSD featuring a solar air collector equipped with a box‐type absorber. The system achieved a maximum efficiency of 60.5%, surpassing the efficiencies of flat and finned solar collectors by 39.5% and 24.5%, respectively. Navale et al.^[^ ^43^ ^]^ constructed a cabinet‐type NDSD for drying fenugreek leaves. During testing, the NDSD maintained a maximum temperature differential of ≈20–22 °C compared to the ambient temperature. The average drying efficiency of the NDSD was recorded at 34.50%, which marked a 13.98% improvement over an OSD method.

**Figure 8:**
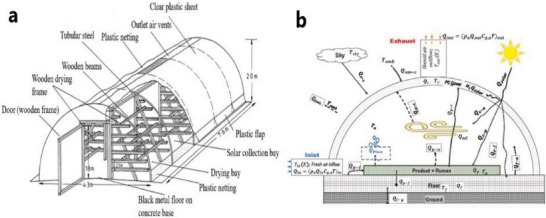
Green house type DSD a. polythene tent dryer Reproduced with permission^[^ ^44^ ^]^ b. solar dome dryer Reproduced with permission © 2021 Elsevier Ltd.^[^ ^45^ ^]^

**Greenhouse DSD**: The drying system (depicted in **Figure** **8**) comprises a structured bamboo framework covered with a transparent polyethene sheet on the side facing the sun and at the lateral ends. The rear side is clad with a black polyethene sheet, extending to the floor to enhance solar radiation absorption. One end of the cladding is designed for human access to the drying chamber. A transparent plastic cover along the bottom edge of the front side wraps around a bamboo pole, adjustable to regulate airflow into the chamber. At the same time, vents positioned at the top ends serve as outlets for humid exhaust air.^[^ ^44^ ^]^ A recent application of a natural convection greenhouse dryer was studied by Colorado^[^ ^45^ ^]^ to reduce the moisture content of biomass waste from 60 to 15%, working under a temperature range of 40 to 70 °C. The dryer is designed in a semi‐cylindrical shape with a capacity of ≈10000 kg. The estimated drying period for the ruminal content was ≈6 days, resulting in a final product moisture content of 14.10%. The tent dryer offers advantages due to its straightforward construction, operational simplicity, and affordability. However, its susceptibility to damage in windy conditions represents a notable drawback.^[^ ^46^ ^]^ A few of the novel DSDs are tabulated in **Table** **2**

**Table 2:** Direct Type NDSD Mode.

Author	Crops Dried	Objective	Outcome/Observation
Kabeel et al.^[^ ^47^ ^]^	Anchovy fish	Using an external reflector, an experimental comparison of OSD and cabinet dryer was done using natural convection.	A 20% energy savings was seen using a modified solar dryer with a reflector compared to OSD.
Sharma et al.^[^ ^48^ ^]^	Turmeric rhizomes	Drying quality of turmeric dried by hot air in a DSD.	Energy savings were seen using a modified solar dryer with a reflector.
Camaño et al.^[^ ^49^ ^]^	Red Tilapia	Effect of sample thickness and its location inside a DSD on the drying kinetics.	Higher diffusivity causes lower drying time using DSD.
Elangovan et al.^[^ ^50^ ^]^	Red bananas	Single‐slope DSD.	Computer vision is NDT that can monitor quality in the spice industry.
Souto et al.^[^ ^51^ ^]^	Tomatoes	DSD cabinet type.	Drying by forced convection is more efficient than natural.
Kumar et al.^[^ ^52^ ^]^	Various	DSD Mathematical model	Tomatoes “Carmen” dried within 30 h.
Sharma et al.^[^ ^53^ ^]^	Turmeric rhizomes	Effect of DSD attributes of turmeric using computer vision.	Drying rates and efficiency decreased with the number of drying days.

**Figure 9:**
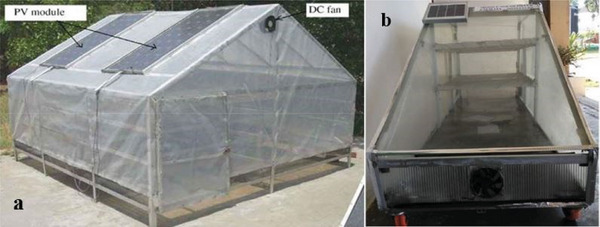
a. Schematic diagram of forced convection solar dryer Reproduced with permission © 2008 Elsevier Ltd.,^[^ ^54^ ^]^ Poratble FDSD for shrimp feed^[^ ^55^ ^]^ Reproduced with permission © 2022, Springer Nature.

Forced convection type direct solar dryer (FDSD): In an FDSD, elevated air temperatures are sustained using a blower or fan to accelerate moisture removal. This solar dryer allows control over chamber temperature and humidity by adjusting airflow through vents. Such control enables the drying of moisture‐rich foods like mushrooms and grapes. Several factors influence the dryer's performance, including ambient temperature, humidity levels, radiation intensity, internal temperature, airflow dynamics, and food density.^[^ ^54^, ^55^ ^]^ **Figure** **9** shows a greenhouse type (Figure 9a) and cabinet type (Figure 9b) FDSD used for commercial and domestic purposes. A few of them are listed in **Table** **3**.

**Table 3:** Direct Type FDSD Mode.

Author	Crops Dried	Outcome/Observation
Peter et al.^[^ ^55^ ^]^	shrimp feed	A portable DSD with an assisted exhaust fan was evaluated. The portable solar dryer was efficient for drying shrimp feed.
Álvarez et al.^[^ ^56^ ^]^	Beef	The drying kinetics of beef drying were evaluated, and data were fitted into five drying models. The beef was dried from 20 kg to 7.5 kg in 11 h. The page model fits the drying behavior of beef best.
Morad et al.,^[^ ^57^ ^]^	Peppermint plants	Three identical tunnel dryers were developed and tested under different conditions, namely different loads, plant conditions, and airflow rates. Continuous fan operating conditions increase the drying rate by 22.78% compared to periodical operating conditions, but overall costs also increase in this case.
Sahdev et al.^[^ ^58^ ^]^	Groundnut	The *h_c_* for the groundnut drying in the greenhouse dryer with different tray size conditions. The value of *h_c_* decreased with the increase in the tray size.
Solomon et al.^[^ ^59^ ^]^	Grapes	The black‐painted solar dryer is more efficient than an OSD.by 2–5 times
Sallam et al.^[^ ^60^ ^]^	Mint	Two identical DSD and ISD were developed to operate in natural and forced convection modes. The faster drying was observed in the case of forced convection drying. The drying behavior in both modes was tested by a curve‐fitting method of 10 thin‐layer drying models.
Rahman et al.^[^ ^61^ ^]^	Egyptian Siwi dates	The DSD cabinet type significantly decreased the levels of lead (Pb) by 96 %, cadmium (Cd) by 94%, copper (Cu) by 48%, nickel (Ni) by 71%, chromium (Cr) by 64%, zinc (Zn) by 4%, manganese (Mn) by 26%, and iron (Fe) by 7% in the dried when compared to OSD.
Kushwah et al.^[^ ^62^ ^]^	banana slices	Initial moisture content (MC) of banana slices was obtained as 78% (wb), decreased to 23.2% (wb) for HX‐evacuated tube‐assisted DSD, 25.6% (wb) by using greenhouse DSD and 28.8% (wb) by using thin‐layer drying kinetics, in 9 h of drying time.
Tiwari et al.^[^ ^63^ ^]^	Jaggery	The value of h_c_ for 800 gm was 1.41 for natural convection and 1.47 W m^−2^K for forced.
Hidalgo et al.^[^ ^64^ ^]^	green onions	DSD solar dryer was tested under natural and forced convection with The effective diffusivity of 5.15 × 10^−9^ m^2^ s^−1^ for natural and 1.15 × 10^−8^ m^2^ s^−1^ for forced convection.
Philip et al.^[^ ^65^ ^]^	Tomato, carrot, bitter gourd	FDSD green house dryer with PV driven exhaust fan. Raised inner temperature by 10–14 °C higher than ambient temperature. Moisture content reduced to ≤5%. Drying time is reduced when compared to OSD.

#### 2.1.2 Indirect Solar Drying (ISD)

**Figure 10:**
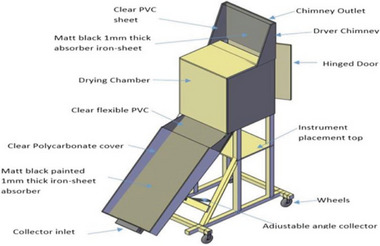
Indirect solar dryer. Reproduced with permission © 2016 Elsevier Ltd.^[^ ^66^ ^]^

**Figure 11:**
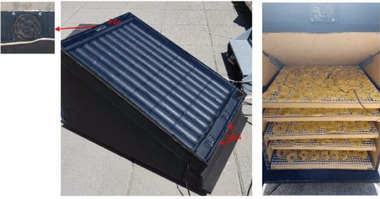
Experiment setup with solar collector module and an exhaust fan for drying apple slices Reproduced with permission ©MDPI.^[^ ^67^ ^]^

In ISD, solar radiation strikes a separate solar collector, which heats the air inside. This heated air is transferred to the drying chamber to extract moisture from the products. The drying chamber is typically constructed from opaque or solid materials to shield the products from direct sunlight exposure. An example of such a dryer was developed and analyzed by Musembi, Kiptoo, and Yuichi,^[^ ^66^ ^]^ depicted in **Figure** **10**. This dryer included a flexible solar collector comprising a transparent polycarbonate cover and an iron‐sheet absorber, a drying chamber equipped with multiple drying trays, and a chimney featuring a metallic absorber plate designed specifically for drying fresh apple slices measuring 2.5 mm thick. Fernandes et al.^[^ ^67^ ^]^ developed and evaluated two ISD prototypes tailored for fruits and vegetables, as seen in **Figure** **11**. Each dryer comprised a solar panel and a drying chamber, with Prototype 1 constructed primarily from wood and Prototype 2 made from styrofoam, both incorporating recycled aluminum cans. Solar panel efficiencies were calculated at 82% for Prototype 1 and 77% for Prototype 2. Results showed that the solar dryers achieved moisture removal rates of 95.7% and 95.0% for Prototype 1 and Prototype 2, With dried product cost was 6.83 € kg^−1^ for the electric dryer, 1.78 € kg^−1^ for Prototype 1, and 1.72 € kg^−1^ for Prototype 2. The novel solar dryers effectively produce high‐quality dried products at significantly lower costs over their operational lifespan, enabling competitive performance against electric systems in reducing food waste economically and sustainably.

**Figure 12:**
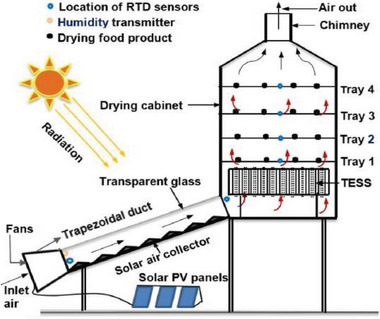
Schematic of Solar dryer with TES arrangement Reproduced with permission © 2023, Springer Nature.^[^ ^68^ ^]^

Gilago et al.^[^ ^68^ ^]^ evaluated passive and active convection ISD systems employing thermal storage systems (paraffin wax) during the drying of carrot slices. Initially, a passive setup (Type‐I) was developed and subsequently upgraded to an active configuration (Type‐II) with solar‐powered fans to enhance mass flow rates, as seen in **Figure** **12**. Comparative analysis revealed improvements in several critical parameters with Type‐II over Type‐I: actual heat supply increased by 11.8%, activation energy by 12.2%, and specific energy consumption by 20.7%. Collector efficiencies averaged 59.7% for Type‐I and improved to 67.8% for Type‐II, while drying efficiencies rose from 11.1% to 14.2%, indicating 13.6% and 27.93% improvements, respectively. Type‐II also demonstrated enhancements in coefficients of moisture diffusion (D_e_), heat transfer (h), mass transfer (h_m_), and specific moisture extraction rate by 20.83, 16.9, 14.52, and 27.8% compared to Type‐I. The moisture content decreased from 9.13 to 0.478 kg kg^−1^ of dry basis in 15 h with Type‐I and 12 h with Type‐II, achieving a faster drying rate and saving 3 h of drying time in Type‐II. A few indirect‐type solar dryers are tabulated below in **Table** **4**.

**Table 4:** Noticeable published work employing ISD techniques.

Author	Principle of Study	Crop/Product	Principal Achievements
Fernandes et al.^[^ ^67^ ^]^	Recycled materials were used, which are environmentally friendly and low production costs for home‐made snacks.	Various	Cost‐benefit when compared electric dryers with moisture removal of 90–95%
Gilago et al.^[^ ^68^ ^]^	Comparison of drying performance of ISD with and without TES in natural and forced air circulation.	Carrots slices	Forced convection outperformed natural convection in all parameters.
Krabch et al.^[^ ^69^ ^]^	ISD with single compartment inclined at 50° and 34°.	Pears	Thermal and economic performances of the designed dryer
Noori et al.^[^ ^70^ ^]^	Solar collector integrated ISD compared with OSD systems	Apples	The effectiveness of ISD with solar PV was higher compared to OSD
Lingayat et al.^[^ ^71^ ^]^	ISD has a solar absorber plate with triangular roughness.	Tomato and brinjal	drying kinetics of brinjal and tomato improved.
Noutfia et al.^[^ ^72^ ^]^	Comparison of ISD and OSD	Figs	drying time deduced from 10 to 4 days.
Etim et al.^[^ ^73^ ^]^	Effect of air intake geometry (square, rectangular, circular, and triangular) on drying kinetics.	Banana	The dryer efficiency was maximum for the triangular‐shaped inlet, having a 20 cm^2^ area.
Moghimi et al.^[^ ^74^ ^]^	Indirect FCSD was developed and tested experimentally and numerically	Tomato	Overall efficiency was 16.4% with a maximum temperature of 65 °C

Solar dryers typically require significant capital investment, making their financial viability crucial for competing with other commercial drying systems. A financial evaluation generally considers the initial cost of the dryer (fixed cost), operating costs, and the payback period. Solar dryers can be cost‐effective if fuel savings offset the additional investment for the solar system or if the overall equipment cost is reduced. Designers or users of solar dryers aim to optimize the balance between cost, energy efficiency, product quality, and the final price. Payback refers to the time (in days, months, or years) needed to recover the total investment through operational cash inflows. Utilizing the dryer year‐round, rather than just seasonally, can lower drying costs and the payback period. Economic analysis of solar dryers should also consider benefits such as improved product quality, higher yields, smaller required space, and faster drying times. A solar dryer with a 50 kg capacity typically ranges from Rs. 30 000 to Rs. 50 000. At the same time, larger industrial‐scale systems may cost between Rs. 4 lakhs and Rs. 10 lakhs, according to a FICCI report. A breakdown of the costs for various types of solar dryers is provided in **Table** **5**.

**Table 5:** Different solar dryers with their cost.^[^ ^75^ ^]^

Types of solar dryer	Initial investment [Rs.]	Operating and maintenance cost per year [Rs.]	Life [Year]
Greenhouse dryer	39 000	200	30
Solar cabinet dryer	5000	200	5
Hybrid PVT integrated	43 000	1000	35
Solar cabinet dryer	5000	200	5

### 2.2 Hybrid Solar Dryer (HSD)

From the above study, the solar drying technology(direct and indirect) represents a sustainable and eco‐friendly approach to food processing, offering potential benefits such as reduced post‐harvest losses, enhanced food quality, lower energy expenditures, and environmental mitigation. However, several challenges are associated with the direct and indirect type are listed below^[^ ^76^ ^]^:

1. Awareness and Education: Farmers and consumers lack awareness about the benefits and proper implementation of solar drying.
2. Initial Cost: High upfront costs for purchasing and installing solar drying systems can hinder adoption, particularly for small‐scale farmers.
3. Maintenance: Continous maintenance requirements, cleaning solar collectors, checking for leaks, and ensuring proper airflow can be challenging and require technical expertise.
4. Weather Dependence: Variability and unpredictability of solar radiation and weather conditions can impact the performance and reliability of solar dryers, affecting drying efficiency.
5. Moisture Control: Difficulty in maintaining consistent moisture levels in dried products, which can affect quality and shelf‐life.
6. Quality Standard: Lack of standardized processes and quality control measures for solar‐dried products necessary to meet market expectations and regulatory requirements.
7. Energy Storage: There are limited options for energy storage in solar dryers, which can affect their ability to operate effectively during periods of low or no sunlight.
8. Scaling and Integration: Challenges in scaling up solar drying systems for larger production volumes and integrating them with existing food processing infrastructure.
9. Seasonality: Seasonal variations in solar radiation and drying conditions can affect solar dryers' year‐round usability and efficiency.
10. Technical Expertise: Technical expertise and training are required to properly install, operate, and troubleshoot solar drying systems.

Among the challenges, a crucial factor affecting solar dryers' performance is their dependence on weather/climate and energy storage. To address such issues, an HSD has recently gained popularity, which can help promote broader adoption and effective utilization of solar drying in various sectors.

**Figure 13:**
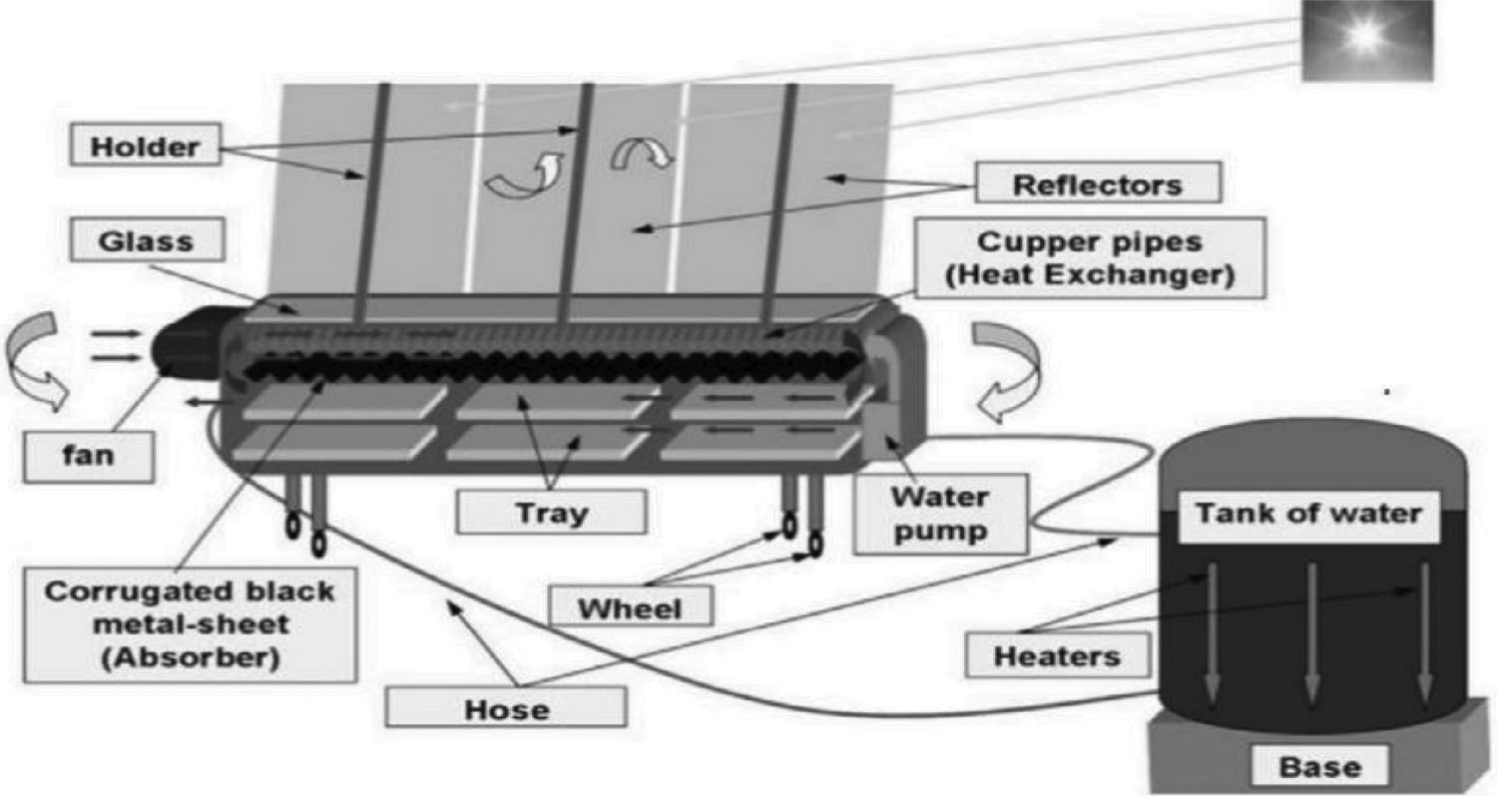
Schematic diagram of hybrid solar dryer Reproduced with permission © 2024 Elsevier Ltd.^[^ ^77^ ^]^

A HSD is a drying system that integrates solar energy with another energy source, such as biomass, LPG (liquefied petroleum gas), electricity, or waste heat, to enhance drying efficiency and reliability. The hybrid approach aims to overcome the limitations of traditional solar dryers, particularly their dependency on sunlight availability, as shown in **Figure** **13**.^[^ ^77^ ^]^

**Figure 14:**
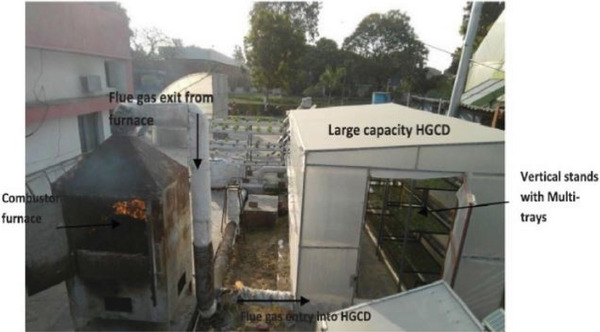
Picture of a multi‐tray, high‐capacity HGCD using paddy straw biomass bales as fuel. Reproduced with permission © 2020 Elsevier Ltd.^[^ ^78^ ^]^

A novel innovation termed the hybrid greenhouse crop dryer (HGCD), integrates solar energy and biomass heat for crop drying within a greenhouse environment, illustrated in **Figure** **14**. This system operates continuously at a consistent temperature of 62 °C throughout the day. A mathematical model was devised to optimize its performance to calculate the biomass heating needs, accounting for forced air circulation and radiant heat transfer.

**Figure 15:**
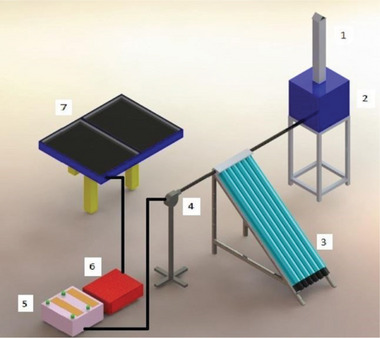
Schematic of noval hybrid drying technique Schematic diagram of ETC aided forced convection Solar Dryer. Label: 1. Chimney, 2. Drying Chamber, 3. ETC, 4. Blower, 5. Battery, 6. Inverter, 7. Photovoltaic (PV) Panel. Reproduced with permission © 2021 International Solar Energy Society. Published by Elsevier Ltd.^[^ ^80^ ^]^

Financial analysis indicates that this solution becomes profitable within five years. Moreover, it offers potential benefits for managing rice straw in India, as highlighted in research by Sethi and Dhiman.^[^ ^78^ ^]^ Asnaz et al.^[^ ^79^ ^]^ compared three cost‐effective solar dryer types, NDSD, FDSD, and HSD, focusing on drying experiments with mushroom slices. The study aimed to analyze drying characteristics for different slice thicknesses, assess the impact of pretreatment on drying times, and compare collector efficiencies. Findings revealed that slicing mushrooms thinly reduced drying time by an average of 40.83 min, while pretreatment shortened drying duration by 26.66 min, equating to a 7% improvement overall. Thermal efficiencies averaged 59.74% for NDSD, 67.66% for FDSD, and 77.45% for HPD. Veeramanipriya and Umayal Sundari^[^ ^80^ ^]^ developed a hybrid PV thermal solar dryer incorporating an evacuated tube collector engineered to expedite cassava drying. Experimental results indicate that the drying time for cassava, starting with an initial moisture content of 91.5% was reduced to just 8 h to achieve an equilibrium moisture content of 10.67% (**Figure** **15**). In comparison, traditional OSD required 13 h to achieve similar results. Ferrari et al.^[^ ^81^ ^]^ proposed to combine kiln dryers for processed timber, utilizing solar and biomass energy sources. Heating was provided by solar collectors and a supplementary biomass furnace fueled by wood waste from a nearby sawmill. Photovoltaic (PV) panels also generate electricity for ventilation and other electrical devices. Climate control was managed by actuators controlled via an electronic system similar to those used in industrial kiln dryers. Initial tests demonstrated enhanced drying capabilities compared to ambient conditions, reducing drying times and moisture content. The data underscored that biomass furnaces increased the drying rate from 0.4% to 1.4% daily. Energy costs were estimated at $0.009 per kWh for the furnace, $0.05 for the solar collector, and $0.14 for the PV system.

A few recent works on HSD have been tabulated in **Table** **6**.

**Table 6:** Compound type/hybrid type.

Author	Principle of Study	Crop/Product	Principal Achievements
Deef et al.^[^ ^82^ ^]^	HSD, with solar collector, assisted drying chamber, and auxiliary air heating system using pumped water from the heating tank.	Fish Waste Processing	maximum of 1.41 kgH_2_O kg^−1^ drying rates at 55 °C.
Reza et al.^[^ ^83^ ^]^	Cabinet‐type HSD with PV module and PCM	Fish	PV module is used in the daytime while PCM transfers energy in the night
Murugesan et al.^[^ ^84^ ^]^	Solar dryer coupled with waste heat dryer using AC unit	Banana slices	The exergetic efficiency was 45 %.
Suherman et al.^[^ ^85^ ^]^	In HSD, air is preheated using LPG and supplied to a solar dryer.	Sliced lime	An average efficiency ranged between 5.36% to 38.61%.
Hou et al.^[^ ^86^ ^]^	Solar‐assisted with multi‐source heat pump(SMSHP) drying system	Multiple	Higher performance was seen in spring, where the SMSHP showed a 22.69% rise in COP and a 19.2 % rise in specific moisture extraction rate, with a 16.11 % decrease in energy consumption.
Rizal et al.^[^ ^87^ ^]^	Solar dryer with air heating running on biomass	Fish	Dry fish in 15 h
Sharma et al.^[^ ^88^ ^]^	Indirect‐type domestic HSD	Tomatoes	Dryer exergy investigation, drying kinetics, and performance evaluation. Moisture content reduced from 95% to 9wt.% in 10 h.

## 3 Advanced Hybrid Solar Dryer

**Figure 16:**
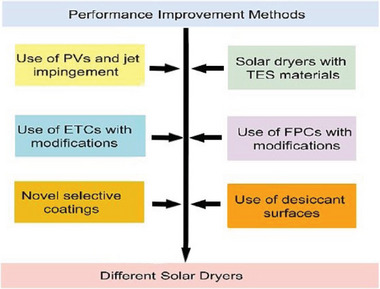
Performance enhancement methods used in solar drying systems.

The efficiency of solar dryers typically hinges on their configuration, solar radiation intensity, air properties, and the initial moisture content of the dried materials. Various strategies have been employed to enhance performance across different dryer designs, including the use of solar collectors, reflectors, surface coatings, jet impingement systems, multiple airflow paths through collectors, integration of desiccant wheels, nanofluids, and applications of energy storage materials^[^ ^89^, ^90^, ^91^ ^]^ **Figure** **16**. These method aimed to reduce energy expenditures and preserve product quality throughout the drying process.

### 3.1 Thermal Energy Storage (TES)

TES is an excellent remedy because solar dryers are restricted from operating solely during daylight hours. In periods without sunlight, microbial growth may occur, potentially leading to the proliferation of microorganisms, degrading agricultural products' quality, causing diminished product quality and spoilage. TES entails storing and preserving thermal energy for later use. This technology is divided into sensible (SHS), latent (LTES), and thermochemical energy storage.^[^ ^92^, ^93^ ^]^

In SHS, the temperature of the storage medium fluctuates within the specified application temperature boundaries without undergoing any phase change. In contrast, LTES involves the storage material experiencing a phase transition, such as solid‐to‐liquid or vice versa, in addition to temperature variations. Thermochemical energy storage relies on the storage and release of heat through reversible chemical reactions.

**Figure 17:**
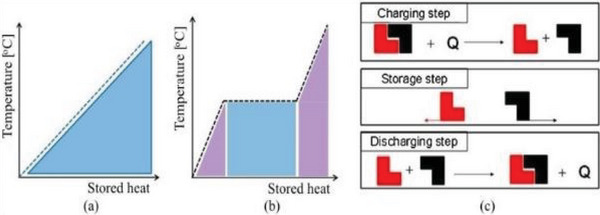
Various methods of TES: (a) sensible heat; (b) latent heat; (c) thermo‐chemical. Reproduced with permission © 2015 Elsevier B.V.^[^ ^95^ ^]^

The relationship between capacity, capability, and discharge duration is intertwined. In specific storage setups, the capacity and capability can influence each other. **Table** **7** ^[^ ^94^ ^]^ presents standard parameters for TES systems, encompassing capacity, capability, efficiency, duration of storage, and expenses. A storage system's optimal characteristics include high‐density energy storage and robust power capability for charging and discharging processes. It is widely recognized that there exist three techniques for TES across temperatures ranging from −40 °C to over 400 °C: sensible heat, latent heat utilizing phase change materials (PCMs), and thermo‐chemical heat storage (TCS) involving chemical reactions (**Figure** **17**).^[^ ^95^ ^]^

**Table 7:** Typical parameters of TES systems.^[^ ^94^ ^]^

TES System	Capacity [kWh/t]	Power [MW]	Efficiency [%]	Storage Period	Cost [€/kWh]
Sensible (hot water)	10–50	0.001−10.0	50–90	days/months	0.1–10
PCM	50–150	0.001−1.0	75–90	hours/months	10–50
Chemical reactions	120–250	0.01−1.0	75–100	hours/days	8–100

The selection of storage media varies depending on the specific application. For heating water, storing energy as the sensible heat of water is a practical choice. When utilizing air‐heating collectors, storage options may include sensible or latent heat effects within particulate storage units, such as in a pebble‐bed HX. Passive heating systems typically store energy as sensible heat within building components. TCS emerges as a suitable option for processes involving PV/photochemical methods.

TES using sensible heat (SHS) (Figure 17a) involves storing thermal energy by heating/cooling a liquid/solid storage medium, such as molten salts, sand, water, or rocks. Water is the most economical option widely used in residential and industrial settings. Sensible heat storage in liquid and solid media underground is also employed for large‐scale applications. SHS offers two primary advantages: cost‐effectiveness and the absence of hazard linked with toxic materials. The SHS system leverages the storage medium's heat capacity and temperature variation during charging and discharging. The stored heat quantity is governed by the quantity of storage material, the specific heat capacity of the medium, and the temperature change.^[^ ^96^ ^]^

**Table** **8** presents a compilation of commonly utilized SHS materials and their properties.^[^ ^97^ ^]^ Water is the premier liquid for SHS due to its affordability and high specific heat capacity. However, for temperatures exceeding 100 °C, oils, molten salts, and liquid metals are preferred alternatives. Storage materials like rock beds are commonly employed in applications involving air heating.

**Table 8:** List of selected solid‐liquid materials for SHS.^[^ ^97^ ^]^

Medium	Fluid type	Temperature Range [°C]	Density [Kg/m^3^]	Specific Heat [J/[Kg.K]
Rock	–	20	2560	879
Concrete	–	20	2240	880
Aluminium	–	20	2707	896
Water	–	0–100	1000	4190
CalorieHT43	Oil	12–260	867	2200
Ethanol	Organic Liquid	≤78	790	2400
Butane	Organic Liquid	≤118	809	2400
Isopentanol	Organic Liquid	≤148	831	2200

**Table** **9** presents the primary characteristics of widely employed solid‐state materials for thermal storage,^[^ ^98^ ^]^ encompassing sand‐rock minerals, fire bricks, ferroalloy materials, and concrete. These materials operate within temperatures ranging from 200 – 1200 °C and exhibit notable thermal conductivities: 1.0 to 7.0 W (m·K)^−1^ for sand‐rock minerals, concrete, and fire bricks and 37.0 to 40.0 W (m·K)^−1^ for ferroalloy materials. The materials detailed in Table 6 are all economically viable, priced between $0.05 and $5.00 per kilogram. Their primary drawback lies in their relatively modest heat capacities, spanning from 0.56 to 1.3 kJ (kg⋅°C)^−1^, potentially necessitating impractically large storage units.

**Table 9:** Solid‐state sensible heat storage materials.^[^ ^98^ ^]^

Storage Materials	Working Temperature [°C]	Density [kg/m^3^]	Thermal Conductivity [W/[m⋅K]]	Specific Heat [kJ/[kg⋅°C]]
Sand‐rock minerals	200–300	1700	1.0	1.30
Reinforced concrete	200–400	2200	1.5	0.85
Cast iron	200–400	7200	37.0	0.56
NaCl	200–500	2160	7.0	0.85
Cast steel	200–700	7800	40.0	0.60
Silica fire bricks	200–700	1820	1.5	1.00
Magnesia fire bricks	200–1200	3000	5.0	1.15

PCMs, known as LTES materials, can absorb or release energy as they change physical state. This characteristic allows for an increase in energy storage density and a corresponding reduction in volume (Figure 17b). During the phase‐change process, heat is primarily stored at a nearly constant temperature directly associated with the latent heat of the substance. Utilizing LTES systems with PCMs represents an efficient TES method, offering high‐energy storage density and an isothermal storage process.

A significant advantage of LTES systems over SHS is their capability to store heat within a similar temperature range. Initially, these materials behave similarly to SHS materials, with temperature rising linearly in response to system enthalpy. However, heat is absorbed or released during phase change at nearly constant temperature, marking a distinct advantage in thermal storage applications.

The choice of a suitable PCM for an LTHS system is based on an evaluation of its various characteristics, including cost, volume expansion, thermal conductivity, latent heat of fusion, subcooling, stability, melting point, and composition.^[^ ^99^ ^]^

PCMs are categorized based on their composition into organic, eutectic, and inorganic materials, as seen in **Table** **10**. Furthermore, these compositional categories can be further subdivided based on melting temperature range, including low‐temperature PCMs (melting below 120 °C), medium‐temperature PCMs (melting between 120 and 300 °C), and high‐temperature PCMs (melting above 300 °C).^[^ ^100^ ^]^

**Table 10:** Commonly used PCMs employed in TES arrangements.

Group	Properties	Subgroup	PCM	No.
	Inert to corrosion Supercooling is negligible Stable in the laboratory that leads to maintaining long‐term functionality, Flammability is detrimental to firefighting clothes.		Microtek MPCM	[101, 102]
Organic		Paraffin	Rubitherm RT‐Line	[103, 104]
			n‐Octadecane	[105, 106]
			PCM Products PlusICE Organic Range	[107, 108]
			Stearic acid	[109, 110]
			n‐Eicosane	[111, 112]
			Lauric acid	[113, 114]
		Non‐ paraffin	Organic Mixture PCMs(fatty acids, polyethylene glycol (PEG), polyalcohol, and polyethylene)	
			OM29 OM37, OM42, OM55	[115, 116]
			Biobased PCMs	[117]
			Coconut oil	[118, 119]
Inorganic	Because of breakdown (phase separation), a thickening and nucleating agent is required. Corrosion, Supercooling	Metallic Salt hydrate	Cited review for comprehensive information regarding metallic materials	[120]
			PCM Products PlusICE Hydrated Salt (S) Range	[121, 122]
			NaNO_3_	[123, 124]
Eutectics	Insufficient thermo‐physical property test data is currently available.		Cited review for comprehensive information regarding eutectic materials.	[125]

Despite the benefits of PCMs in various TES applications, such as solar drying, preparing PCMs that are thermally and chemically stable while retaining desired properties remains a significant challenge. Researchers have recently pioneered innovative methods for synthesizing PCMs to enhance their long‐term storage capabilities to address this. These advancements encompass a range of techniques, including PCM slurries, composite PCMs featuring dual phase transition temperatures, shape‐stabilized PCMs utilizing microencapsulation, nano‐confinement of organic PCMs, PCMs constructed from metal–organic frameworks (MOFs), and pioneering strategies like incorporating photo‐switching dopants to manipulate PCM behavior.^[^ ^126^ ^]^

Shape‐stabilized PCMs are fabricated through microencapsulation, employing a method where PCM particles disperse within a conventional carrier fluid, thereby encapsulating the PCM within a small, sealed container that prevents liquid PCM leakage. This approach enhances thermal cycling durability, maintains a consistent volume, and improves heat transfer surface area for TES.^[^ ^127^ ^]^ Various shell materials are utilized for microencapsulating diverse PCMs,^[^ ^128^ ^]^ encompassing organic, inorganic, and organic–inorganic hybrid types. Examples of inorganic shell materials include acrylic resin, silica, and zinc oxide, while organic–inorganic hybrid materials like polymethyl methacrylate (PMMA)‐SiO_2_ and PMMA‐TiO_2_ are also employed. A few of the recent works have been tabulated in **Table** **11**.

**Table 11:** Solar Dryer using LTES.

Author	Principle of Study	Crop/Product	Principal Achievements
Kannapiran et al.^[^ ^129^ ^]^	Comparisons between OSD, ISD with and without PCM	Mango slices	moisture content reduced from 84% to 25% in 10h.
Çakmak & Yıldız^[^ ^130^ ^]^	Novel swirl generator for increasing turbulence and PCM‐assisted drying	Grape	Final drying levels are achieved in 56 h, which takes 200 h in an OSD.
Gilago et al.^[^ ^131^ ^]^	Comparison of ISD under natural and forced circulation assisted with PCM	Pineapple	Dryer efficiency improved by 25.77 % and collector by 16.52 % in forced convection ISD.
Panchal et al.^[^ ^132^ ^]^	Multiple PCM (cascaded in rectangular chambers) in an ISD	Potato slices	2 h faster drying time in comparison to ISD without PCM. Also, 1.13 kg of water evaporated using PCM in comparison to 0.87 kg using conventional ISD.
Román et al.^[^ ^133^ ^]^	Effect of LHTS size in the drying time.	Wheat	PCM reduced the drying time by −5% to +13.9%
Arunkumar et al.^[^ ^134^ ^]^	IDS under natural convection using PCM with glass pieces	red chilli	Moisture content dropped from 83.8% to 7.3% in 72 h.
Lad et al.^[^ ^135^ ^]^	Effect of changing PCM location in the absorber or dryer.	various	A dryer integrated with PCM gives maximum performance.
Bareen et al.^[^ ^136^ ^]^	IDS under forced convection with solar collector with PCM	Cymbopogon citratus	Using PCM, drying air temperature was higher by 9–12 °C after sunset for 4h.

Chokngamvong et al.^[^ ^137^ ^]^ used OpenFOAM to simulate the hot‐air drying kinetics of pineapple slices. The developed model integrates conjugate heat and moisture transfer, combining forced convection phenomena with moisture diffusion dynamics. Modifications to the original OpenFOAM solver using C++ programming enable accurate heat and moisture transfer representation via the finite‐volume method, giving an average R^2^ of 0.9443. The SST k–ω turbulence model also effectively characterizes airflow around the pineapple slice model. The model's predictions align closely with experimental findings, demonstrating an average error of less than 1.29%. Further analysis using the model reveals insights into how variations in pineapple slice geometry—such as increased inner diameter and reduced thickness—enhance drying efficiency by indices of 1.002 and 1.005, respectively.

TCS employs thermo‐chemical substances, which can store and release thermal energy through a reversible reaction that alternates between absorbing and releasing heat (see Figure 17c). In the charging phase, heat is supplied to substance A, causing it to split into two components, B and C. These resulting reaction products are straightforward to separate and can be stored until needed for the discharge cycle. When required, components B and C are combined under appropriate pressure and temperature conditions, resulting in the release of energy.

The separated products B and C can be stored independently, and heat losses from the storage units are primarily limited to sensible heat effects, which are typically minor compared to the heat released during the reaction.

Research has explored the thermal breakdown of metal oxides for energy storage.^[^ ^138^ ^]^ These reactions offer potential advantages, such as utilizing evolved oxygen for other purposes or discarding it and incorporating atmospheric oxygen into reverse reactions. An example is the decomposition of potassium oxide at a temperature of 300–800 °C with a heat release equivalent to ≈2.1 MJ kg^−1^ and that of lead oxide at a temperature of 300–350 °C with a heat release of 0.26 MJ kg^−1^

(1) $$4KO_{2}↔2K_{2}O+3O_{2}$$

(2) $$2\mathrm{Pb}O_{2}↔2\mathrm{PbO}+O_{2}$$

Fujii et al.^[^ ^139^ ^]^ extensively studied the Ca(OH)_2_. The forward reaction takes place at ≈450 °C. Further reaction rate can be enhanced by zinc or aluminum. CaO obtained using this is kept in the absence of water.

(3) $$\mathrm{Ca}{\left(\mathrm{OH}\right)}_{2}↔\mathrm{CaO}+H_{2}O$$

**Table** **12** ^[^ ^140^ ^]^ shows various chemical reactions particularly notable for TES applications. These reactions can indeed involve much higher temperatures than those typically used for sorption processes.

**Table 12:** Some chemical reactions for TES.^[^ ^140^ ^]^

	Reaction	Temperature [°C]	Energy Density [kJ/kg]
Methane steam reforming	CH_4_ + H_2_O = CO + 3H_2_	480–1195	6053
Ammonia dissociation	2NH_3_ = N_2_ + 3H_2_	400–500	3940
Metal hydrides Thermal dehydrogenation	MgH_2_ = Mg + H_2_	200–500	3079 (heat) 9000 (H_2_)
Metal hydride's thermal dehydrogenation	MgH_2_ = Mg + H_2_	200–500	3079 (heat) 9000 (H_2_)

### 3.2 With Auxiliary Unit

The solar dryer functions primarily on solar energy; however, additional heating is achieved using supplementary units. The most prevalent supplementary units utilize fossil fuels or biomass to attain and sustain the requisite temperatures. Despite their effectiveness, these units are limited in availability and raise concerns related to environmental pollution. Amer and Gottschalk^[^ ^141^ ^]^ employed electric resistances as supplemental units in the drying of fresh chamomile; Matouk et al.^[^ ^142^ ^]^ utilized them for drying onion slices; and Hossain et al.^[^ ^143^ ^]^ employed them for drying tomato slices.^[^ ^144^ ^]^ Ferreira et al.^[^ ^145^ ^]^ utilized 20 incandescent lamps, each 100 W, for drying banana slices. Suherman et al.^[^ ^146^ ^]^ utilized stainless steel (SUS) plates as solar radiation collectors and an LPG (liquefied petroleum gas) burner for drying seaweed. Numerous studies have explored such dryers across various applications.^[^ ^147^ ^]^ Malakar et al.^[^ ^148^ ^]^ focussed on developing and evaluating the performance of an infrared‐assisted HSD designed for drying pineapple slices, which incorporates an evacuated tube solar collector, a blower assembly, a drying chamber, an infrared heater, and a PCM chamber. During experimentation, the average temperatures recorded inside the drying chamber were 60.16 °C for direct drying, 57.29 °C for PCM‐assisted drying, and 60 °C for PCM + IR‐assisted drying. The drying rate observed in PCM + IR‐assisted drying surpassed that of direct and PCM‐assisted methods with moisture diffusivity of 2.59 × 10^−9^ m^2^ s^−1^.

A few remarkable works are tabulated below in **Table** **13**.

**Table 13:** Solar Dryer with auxiliary unit.

Author	Principle of Study	Crop/Product	Principal Achievements
Suherman et al.^[^ ^149^ ^]^	HSD assisted with the LPG burner	Seaweed	Higher temperature causes faster drying. Also, drying fast in the initial phase then reduces. At 60 °C, the drying rate increases by 7.8 times.
Yuwana et al.^[^ ^150^ ^]^	HSD assisted with biomass	fish drying	14.37 h was needed using ISD with biomass, and 15.43 h was needed when operating with solar and continued biomass energy to reduce moisture by 80%.
Partheeban et al.^[^ ^151^ ^]^	Solar‐LPG hybrid dryer	geopolymer bricks	The geopolymer bricks' moisture content reduced from 20% to 0% in 10 h.
Lamrani et al.^[^ ^152^ ^]^	ISD solar electrical dryer	wood	The dryer temperature was increased by 4–20 °C, and the drying time was reduced by 39% compared to the dryer system without the LHTES system.
Suvanjumratet al.^[^ ^153^ ^]^	ISD forced convective solar dryer with electrohydrodynamic	Pineapple slices	A correlation between the corona voltage and the diffusion coefficient was identified. Solar‐EHD dryer at 10 kV would result in yearly savings of 3304.2 THB.
Mugi et al.^[^ ^154^ ^]^	ISD comparison between natural and forced convection(using PV module).	Beetroot slabs	Forced convection drying time saved by 42.31% compared to OSD
Kalita et al.^[^ ^155^ ^]^	Comparison between biogas and electric‐assisted HSD	Red chilli	A drying efficiency of 25.4% was achieved in Biogas‐assisted HSD. Red chilli moisture content was reduced from 70.2% to 17.7% within 16 hr using electric‐assisted HSD and 14 hr using Biogas HSD.
Azis et al.^[^ ^156^ ^]^	HSD (flat plate)with LPG dryer		The highest solar dryer temperature of 57 °C was achieved.
Amer et al.^[^ ^157^ ^]^	Air heater assisted HSD under forded convection.	Lavender, lemongrass, marjoram, thyme	Energy efficient by 19–36% with good green color retention when dried at 40 °C. HSSD helped save 19–36% of energy consumption.
Singh et al.^[^ ^158^ ^]^	DSD (Greenhouse), with forced air convection, is heated using a heat exchanger with hot water.	tomato	Moisture content reduced from 94.6% (wb) to 10% (wb) in 10 h, with a payback period of 1.73 years.

**Figure 18:**
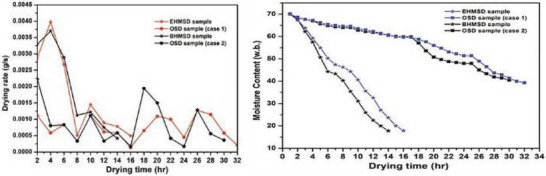
Comperision of performance between different types of HSD Reproduced with permission © 2024 Elsevier Ltd.^[^ ^155^ ^]^

Kalita et al.^[^ ^155^ ^]^ performed a comparison between biogas‐assisted and electro‐hydrodynamic‐assisted HSD showed that biogas‐operated forced convective HSD outperformed electric‐assisted and OSD‐type solar dryers, as seen in **Figure** **18**.

**Figure 19:**
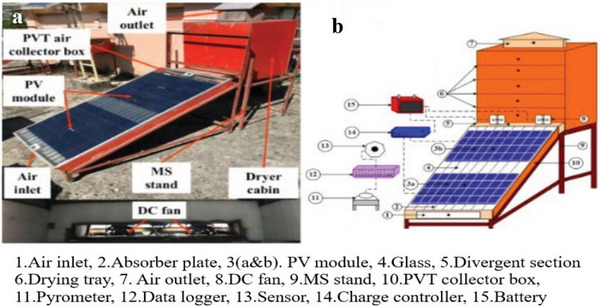
PVT solar HSD; (a) Pictorial view; (b) schematic diagram of the set‐up. Reproduced with permission © 2022 Elsevier Ltd.^[^ ^159^ ^]^

Further, Gupta et al.^[^ ^159^ ^]^ worked on a novel PVT(Photovoltiec‐Thermal) solar drying system for achieving carbon neutrality in the drying operations of star fruit. The novel ISD sustainability metrics were checked through energy and exergy performance alongside environmental and economic evaluations (4E) in both forced convection drying (FCD) and natural convection drying (NCD) modes; the setup is shown in **Figure** **19**. In the PVT solar dryer, the moisture content of star fruit decreases from 10.11% (dry basis) to 0.19% (dry basis) within 12.50 h under FCD and 14.50 h under NCD. Comparative drying under OSD takes 22.00 h. Energy and exergy efficiencies in the PVT system are 69.27% and 31.12%, respectively, under FCD, 43.58%, and 17.89% under NCD. The drying efficiencies are 15.27 and 13.98%, with specific moisture extraction rates of 0.1786 and 0.6657 kg kWh^−1^ and specific energy consumptions of 12.37 and 3.57 kWh kg^−1^ in FCD and NCD modes, respectively.

### 3.3 Desiccant Surfaces/Fins/Surface Coating

Recently, a few researchers have tried to modify the surface in ISD either by surface coating,^[^ ^160^ ^]^ perforation,^[^ ^161^, ^162^, ^163^, ^164^, ^165^, ^166^, ^167^, ^168^, ^169^, ^170^, ^171^, ^172^, ^173^ ^]^ or by extending the solar absorber surfaces with fins,^[^ ^174^ ^]^ roughness,^[^ ^175^ ^]^ turbulators,^[^ ^176^, ^177^ ^]^ using nanoparticles in PCM as HTF (Heat transfer fluids).^[^ ^178^ ^]^ Incorporating fins into a system increases surface area, thereby improving heat transfer between the PCM and the heat source. However, this enhancement comes at the cost of reducing the amount of PCM stored within the same volume, potentially decreasing stored energy capacity. Additionally, fins have the potential to impede natural convection currents, which are critical for efficient heat distribution.^[^ ^179^ ^]^

These conflicting outcomes and challenges underscore the importance of addressing them in future research to optimize the design of LTES systems. Balancing these factors will be crucial in maximizing the efficiency and effectiveness of LHTES technology in various applications.^[^ ^180^ ^]^ A few other recent works have been tabulated below in **Table** **14**.

**Table 14:** Solar Dryer with surface corrugated/surface coating.

Author	Principle of Study	Crop/Product	Principal Achievements
Tuncer et al.^[^ ^160^ ^]^	HSD (greenhouse) is made of soil flour, gravel, and black‐painted and nano‐enhanced black‐painted sheet metal.	–	Sheet metal with nano‐enhanced black paint showed the least drying time.
Rouzegar et al.^[^ ^181^ ^]^	ISD with PV module with V‐surface corrugated collector	Mint	PCM helped in lowering the temperature in the chamber.
Behera et al.^[^ ^182^ ^]^	ISD with PCM in absorber plate and swirl generators	Various	The maximum collector efficiency was 64.23%, while the drying efficiency was 33.61%, and the maximum drying rate was found to be 1.351 kg hr^−1^.
Behera et al.^[^ ^183^ ^]^	ISD with corrugated absorber aluminum plate		maximum absorbing plate temperature reached to89 °C with drying efficiency reached to 43%.
Sethi et al.^[^ ^184^ ^]^	DSD with corrugated surface air heater	tendu leaves	Drying time reduced by 2 h when compared to flat plate dryer
Yüksel et al.^[^ ^185^ ^]^	V‐grooved double pass PVT solar dryer	golden apple slices	Energy efficiency of ISD with LTES varied between 55.11 and 78.47 %.
Khanlari et al.^[^ ^186^ ^]^	Fins were added to the absorber plate with PCM.	——	Porous fin increases the melting time of PCM and attains a thermal efficiency of 41.73%.
Öztürk et al.^[^ ^191^ ^]^	double‐pass solar dryer whose absorber plate is coated with graphene and black paint		Graphene coating DSD showed a thermal efficiency of 73.36%.
Khanlari et al.^[^ ^192^ ^]^	Infrared heating and *Fe* nano embedded paint on perforated absorber plate.		Thermal efficiencies rose by 16.94% and exergetic efficiency by 37.72%.

In recent years, HSD with selective coating has gained popularity. Selective coatings offer innovative means to enhance the efficiency of photothermal exchange in solar energy‐driven drying applications, which is crucial for efficiently gathering heat, especially at elevated temperatures. The absorptance (α) and emissivity (ε) of an absorber characterize its spectral selectivity in the short wavelength and infrared regions, respectively.^[^ ^187^ ^]^ Selective coatings typically exhibit absorptance values ranging from 0.88 to 0.99 and emissivity values from 0.01 to 0.3. Authors generally opt for selective coatings with high absorptance and low emissivity to ensure enhanced collector performance, particularly in various solar drying applications. Tarek et al.^[^ ^188^ ^]^ proposed a black paint based on 4% CNT and copper oxide (CuO) as an exclusive selective coating. This coating demonstrated an absorptance value of 0.964 and an emittance value of 0.124. This led to an efficiency increase of ≈24.4% in the solar dryers studied. Sivakumar et al.^[^ ^189^ ^]^ conducted a similar study, investigating the effects of adding 0.04 vol% CuO nanoparticles to black paint as a selective coating, resulting in a 4% efficiency improvement.

Furthermore, incorporating 1% graphene nanomaterials into black paint significantly enhanced efficiency, raising it from 43.15% to 48.23%. Kabeel et al.^[^ ^190^ ^]^ utilized titanium dioxide (TiO) nanoparticles in black paint to coat the absorber surface. They observed a temperature increase of 1.52 °C in water relative to systems using black paint only on the absorber plate. This approach is promising for enhancing the effectiveness of solar thermal systems. However, selecting appropriate nanoparticles tailored to specific applications is a critical task during the design phase to ensure significant improvements in system performance.^[^ ^178^, ^180^, ^193^, ^194^ ^]^

## 4 Conclusion 

To achieve the established sustainable development objectives and address global challenges related to energy scarcity and food security, adopting strategies for preserving food using renewable energy sources is highly advisable. Solar drying methods are particularly effective in this context due to their various socio‐economic, qualitative, and environmental benefits. The objective of the articles is to consolidate findings from multiple studies, discern patterns within the literature, and offer insights and suggestions for advancing research and development in the realm of solar drying. Additionally, the article thoroughly discusses several techniques to enhance performance, such as employing desiccant surfaces, modifying flat plates, and incorporating TES. Below are summarized some of the critical contributions of this review article.

- The intensity of solar radiation, air velocities, dryer design, and operational modes are critical factors influencing the effectiveness of solar drying systems.
- Forced circulation methods result in quicker drying times and higher quality final products than OSD and NDSD methods, despite limitations in handling more significant quantities of products. When it comes to drying crops on a large scale, the greenhouse method is considered optimal, although it necessitates more space.
- A forced convective dryer is more suitable than a natural convection dryer for products with high moisture content due to its higher drying rate. Incorporating thermal storage allows solar dryers to operate during nighttime and periods of low solar intensity. Integrating solar dryers with energy storage materials, including both sensible and latent heat options, offers a promising solution to mitigate the intermittent nature of solar energy. Research has demonstrated substantial temperature increases and enhanced efficiency through this approach. Studies highlight the effectiveness of sensible heat storage using materials like pebbles, limestone, and beach sand, alongside the dynamic advantages of latent heat storage using PCM such as paraffin. This allows for extended operational periods in remote areas during nights or cloudy seasons.
- This review thoroughly examines recent advancements in research aimed at improving the energy efficiency of solar dryers through various alternative strategies. These strategies encompass hybrid solar dryers that integrate auxiliary heating sources like electric or biomass heaters, solar‐assisted heat pump dryers, the application of desiccant materials, and adopting heat storage systems combining sensible and latent heat storage techniques.
- Positive attributes in terms of quality, environmental impact, and social benefits associated with various solar drying techniques validate their suitability for both household and industrial applications. However, higher costs and, consequently, longer payback periods may limit adoption among small‐scale farmers. Nevertheless, multi‐season usage of these systems can improve payback periods by over 50%.
- Overcoming practical barriers to the market adoption of solar dryers requires addressing system‐level concerns and advancing methodologies. Key challenges include perceptions of limited and intermittent usability of solar dryers and relatively slow drying processes.

## 5 Future Directives

The article has reviewed various solar dryer technologies, yet a research gap remains in identifying an economical and efficient drying system capable of producing high‐quality products. It is evident that the geometric and thermal characteristics of solar dryers significantly influence drying outcomes. Therefore, optimizing these variables can enhance the dryers' performance and the products' quality.

One suggestion is to improve the surface geometry of the absorber, which can potentially increase the heat transfer coefficient within the collector. Additionally, research has shown that utilizing nanofluids and nanoparticles is highly effective for enhancing heat transfer, presenting substantial opportunities for further advancements in this area.

## Conflict of Interest

The authors declare no conflict of interest.

## Author Contributions

The article idea and original draft were conceived by Md.A.R. The literature review and data analysis were carried out by S.M.M.H. and P.P. The revised work was completed by R.Z. and A.G.A.
